# A Systematic Review and Meta‐Analysis of the Impact of *Cornus mas* L. on Anthropometric Indices and Body Composition

**DOI:** 10.1002/fsn3.70404

**Published:** 2025-07-15

**Authors:** Faezeh Ghalichi, Roghayeh Molani‐Gol, Neda Ehsani, Yaser Khajebishak

**Affiliations:** ^1^ Medicinal Plants Research Center, Department of Nutrition Maragheh University of Medical Sciences (MRGUMS) Maragheh Iran; ^2^ Student Research Committee, Faculty of Nutrition and Food Sciences Tabriz University of Medical Sciences Tabriz Iran; ^3^ Student Research Committee, Department of Nutrition Maragheh University of Medical Sciences (MRGUMS) Maragheh Iran

**Keywords:** anthropometric indices, body composition, cornelian cherry, *Cornus mas* L., meta‐analysis, systematic review

## Abstract

Obesity and its related metabolic disorders have been a global concern in health care over the past few decades. Lifestyle modification and evidence‐based dietary interventions, including the usage of nutraceuticals with minimal adverse effects, have gained widespread recognition as effective strategies for obesity management. Cornelian cherry, scientifically identified as *Cornus mas L.* (CM) is rich in anthocyanins, flavonoids, tannins, and polyphenols, with anti‐inflammatory, anti‐oxidant, anti‐diabetic, anti‐obesity, and hypolipidemic properties. Cornelian cherry inhibits hepatic lipogenesis, increases hepatic lipid oxidation and clearance, and regulates the expression of peroxisome proliferator‐activated receptors. Additionally, CM activates the AMP‐activated protein kinase and adiponectin signaling pathways in the white adipose tissue, resulting in adipocytokine level decline. We aimed to perform a systematic review and meta‐analysis of the impact of CM on anthropometric indices and body composition. A comprehensive search was conducted on the Web of Sciences, PubMed, and Scopus databases up to April 2025 for published papers. The quality of methodology and risk of bias of selected studies were assessed by the Cochrane Risk of Bias tool. Overall, six randomized controlled trials (RCT) were included. Pooled analyses indicated no significant differences observed in cases of body weight (BW), body fat percentage (BF%), fat mass (FM), weight circumference (WC) and hip circumference (HC). However, a modest but significant increase in body mass index (BMI) was observed after supplementing CM. Furthermore, according to sub‐group analyses, BW, BMI, BF%, FM, WC, and HC were significantly increased when administration was accomplished on MAPLD patients, the sample size was > 50, the lyophilized dried and CM fruit extract forms were used, intervention duration was < 12 weeks, and doses were ≥ 30 g. Future research should prioritize well‐designed, long‐term follow‐up RCTs investigating CM as an adjunct therapy to multimodal lifestyle interventions, focusing on both anthropometric indices and biochemical markers.

## Graphical abstract

Findings indicated no significant differences in cases of body weight (BW), body fat percentage (BF%), fat mass (FM), waist circumference (WC) and hip circumference (HC) after administering *Cornus mas L*. A modest but significant increase in body mass index (BMI) was observed. According to sub‐group analyses, BW, BMI, BF%, FM, WC, and HC were significantly increased in MAPLD patients, > 50 sample size, with lyophilized dried and CM fruit extract forms, < 12 weeks' intervention duration, and ≥ 30 g doses.

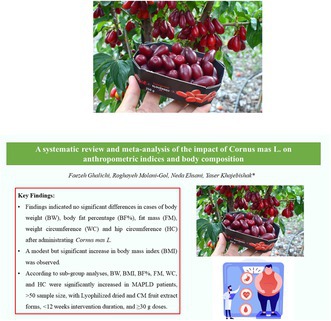

## 1 Introduction

Obesity is considered a complex metabolic disease caused by a positive energy balance and the accumulation of adipose tissue. Epidemiological studies project that approximately 1 billion people will become obese by the end of 2030 (Gallo et al. 2024). Obesity and its related metabolic disorders, such as non‐alcoholic fatty liver disease (NAFLD), metabolic syndrome, and type 2 diabetes mellitus (T2DM), have been a global concern in health care over the past few decades. It is well established that obesity is not only related to higher mortality rates due to cardiovascular diseases (CVD) and hepatic complications but also imposes significant economic burdens on healthcare systems. Anthropometric indices including body weight (BW), body mass index (BMI), and waist circumference (WC) and body composition parameters (e.g., fat mass (FM) and lean body mass) are identified as key factors for assessing the risk of these diseases (Godoy‐Matos et al. 2020; Khajebishak et al. 2023).

Lifestyle modification and evidence‐based dietary interventions including usage of nutraceuticals with minimal adverse effects have gained widespread recognition as effective strategies for obesity management (Musazadeh, Zarezadeh, Ghalichi, Ahrabi, et al. 2022; Musazadeh, Zarezadeh, Ghalichi, Kalajahi, and Ghoreishi 2022). In fact, consumption of natural substances, particularly those with higher contents of phenolics and flavonoids, has been shown to ameliorate metabolic abnormalities and be efficient in weight management (Valizadeh et al. 2019). Recent research highlights that these bioactive compounds, especially phenolic compounds, offer protective effects on the liver, kidneys, and cardiovascular system due to their antioxidant, anti‐allergic, anti‐coagulant, anti‐diabetic, anti‐inflammatory, anti‐obesity, and lipid‐lowering benefits (Asgary, Ghannadi, et al. 2013; Bayram et al. 2024; Dinda et al. 2016).

Cornelian cherry, scientifically identified as *Cornus mas L*. (CM), a member of the Cornaceae family (Koçyiğit and Özhatay 2006), is affluent in bioactive compounds including anthocyanins, phenolic acids, flavonoids (quercetin, kaempferol, and aromadendrin), phenols, organic acids, and tannins (Bayram and Ozturkcan 2020; Dinda et al. 2016; Gholamrezayi et al. 2019). For over a millennium, CM has been utilized as a traditional medicine across central Asia to cure ailments such as T2DM, inflammatory bowel disease, urinary tract infection and fever, malaria, and cancer (Bayram et al. 2024).

Experimental studies on mice models fed with high‐fat diets, alongside anthocyanins and ursolic acid extracted from CM, showed significant improvement in glucose tolerance and body weight gain via reduction in lipid accumulation (Jayaprakasam et al. 2006). Furthermore, the results of experimental meta‐analyses showed significant lipid‐modifying effects for CM (Dayar et al. 2020; Mohammadi et al. 2021). In terms of body composition, the findings of clinical trials are inconsistent. In one study, administration of 900 mg of anthocyanins extracted from cornelian cherry for 8 weeks resulted in significant reductions in BW, BMI, and WC (Gholamrezayi et al. 2019). Conversely, no significant changes were observed for BW, FM, WC, or hip circumference (HC) among NAFLD patients consuming cornelian cherry extract (Yarhosseini, Sangouni, Sangsefidi, et al. 2023). Similar results were also mentioned for T2DM patients (Soltani et al. 2015).

Given the variability of findings and the lack of a meta‐analysis on the association between Cornelian cherry and anthropometric indicators, the current research intended to perform a systematic review and meta‐analysis on the effect of CM on body composition and anthropometric indices.

## 2 Material and Methods

### 2.1 Data Sources and Search Strategy

The present study was based on the Preferred Reporting Items for Systematic Reviews and Meta‐Analyses (PRISMA) guidelines. The research question was structured according to PICOS criteria:

- **P**articipants: Adult men and women.
- **I**nterventions: Administration of CM or cornelian cherry.
- **C**omparisons: Control group.
- **O**utcomes: Anthropometric and body composition indices, including BMI, BW, WC, HC, body fat percentage (BF%) and FM.
- **S**tudy Design: Studies assessing the therapeutic effects of CM or cornelian cherry on anthropometric and body composition indices.

The databases PubMed, Web of Science, and Scopus for peer‐reviewed articles, restricted to studies published in English, were searched up to April 2025 by two independent reviewers (YKB & NE) using the following keywords: “weight OR Body mass index OR BMI OR waist circumference OR WC OR Hip circumference OR HC OR body fat mass OR FM OR body fat percent OR BF% OR fat free mass OR FFM” AND “cornelian cherry OR cherry OR cornelian mass” AND “randomized controlled trial”. The wild‐card term “*” was used for increasing the sensitivity of search strategy. The complete search strategy is detailed in Table S1. Gray literature (e.g., conference abstracts, theses) was excluded due to concerns about incomplete data and lack of peer review.

### 2.2 Study Selection and Inclusion Criteria

Two independent reviewers (FGH, NE) conducted the screening based on title, abstract, and keyword fields for selecting related studies, and then afterward the full texts were assessed thoroughly. Inter‐rater agreement was 92% (*κ* = 0.81), indicating almost perfect agreement. The criteria for including studies were: (a) articles written in the English language; (b) studies assessing the effect of CM or cornelian cherry on body composition and anthropometric indices; (c) placebo and treatment groups were included. Furthermore, studies in other languages, experiments on animal models, in vitro, in vivo, and ex vivo studies, case reports, observational studies, and duplicate papers were not included.

### 2.3 Data Extraction

Based on the primary studies' variables, a standardized data extraction form was developed for selecting and tabulating data (NE & RMG). The data were as following: first author's name and publication year, location, study population, sample size, mean age or age range, supplement form and dose, duration of the intervention, and the mean ± standard deviation (SD) of the body composition and anthropometric indices at the beginning and end of the study.

### 2.4 Quality Assessment

The Cochrane Collaboration's risk of bias tool was used for assessing the methodological quality and risk of bias of selected studies (Minozzi et al. 2020). The Cochrane RoB tool analyzes 10 items related to selection bias (item 1), performance bias (items 2 and 3), detection bias (item 4), attrition bias (item 5), reporting bias (item 6), and other sources of biases (item 7). The Cochrane RoB checklist is categorized into (+) Low risk of bias, (−) High risk of bias, and (?) Unclear risk of bias. The judgment of “yes” indicates low risk of bias, while “no” implies high risk of bias, and labeling an item as “unclear” is considered as unclear or unknown risk of bias. Two reviewers (FGH and NE) evaluated the quality of included studies and any discrepancy was resolved through consensus with a third reviewer (YKB). The certainty of the evidence was assessed using the GRADE approach, with detailed criteria outlined in Table S2.

### 2.5 Statistical Analysis

The pooled effect was calculated using the mean and SD change of BW, BMI, WC, HC, BF%, and FM (difference of the beginning and end of values). In case data were expressed by any value other than mean and SD, standard statistical calculations were employed to estimate the mean and SD, via the following formula: SD difference = square root [(SD_1_^2^ + SD_2_^2^) − (2 × *R* × SD_1_ × SD_2_)]*c*, assuming a correlation coefficient (*R*) of 0.8 as it is a conservative estimate for an expected range of 0–1 (Higgins et al. 2019). *I* ^2^ statistics and the Cochrane *Q* test were used to assess studies heterogeneity (Tarsilla 2010). High between‐study heterogeneity was considered for *I* ^2^ > 50% and *p* < 0.1. For cases with low heterogeneity, the fixed‐effects model was considered; otherwise, the random‐effects model was used.

Analysis of subgroup was conducted for identifying probable sources of heterogeneity and also expressing effect sizes among various subgroups, including population (Insulin resistance, NAFLD), metabolic dysfunction‐associated fatty liver disease (MAFLD), postmenopausal, sex (Female/Male & Female), mean age (≤ 50/> 50 years), supplement form (Lypophilized dried, *C. mas* L. fruit extract, CME capsule), dose (< 30/≥ 30) and intervention duration (≥ 12/< 12 weeks). Weighted mean difference (WMD) and corresponding 95% confidence interval were used to report the overall effect size. If the measurement units were not homogenized, the effect size was expressed as SMD. The leave‐one‐out strategy was used to conduct a sensitivity analysis to see how omitting each study affected the combined effect size. Begg's regression tests were employed for determining the publication bias. Stata 16.0 was used to conduct all statistical analyses (Stata Corp. College Station, Texas, USA).

## 3 Results

### 3.1 Study Selection

**FIGURE 1:**
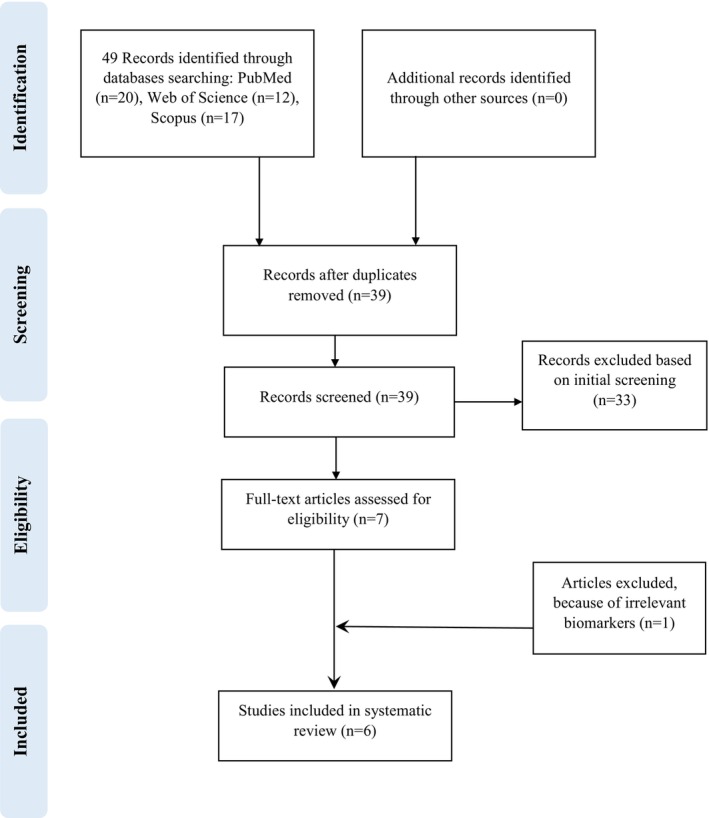
Flow diagram outlining included studies selection.

Based on the flowchart of literature search and selection procedure (Figure 1), a total of 49 records were identified at baseline. After omitting duplicate records, 39 records remained. Of these, 33 articles were excluded after careful review of the abstracts and titles. Afterward, 6 trials were further examined, and 1 trial was excluded due to irrelevant biomarkers. Overall, 6 studies (Asgary, Kelishadi, et al. 2017; Bayram et al. 2024; Celik et al. 2023; Gholamrezayi et al. 2019; Soltani et al. 2015; Yarhosseini, Sangouni, Sangsefidi, et al. 2023) were ultimately included in this systematic review and meta‐analysis.

### 3.2 Studies Characteristics

**FIGURE 2:**
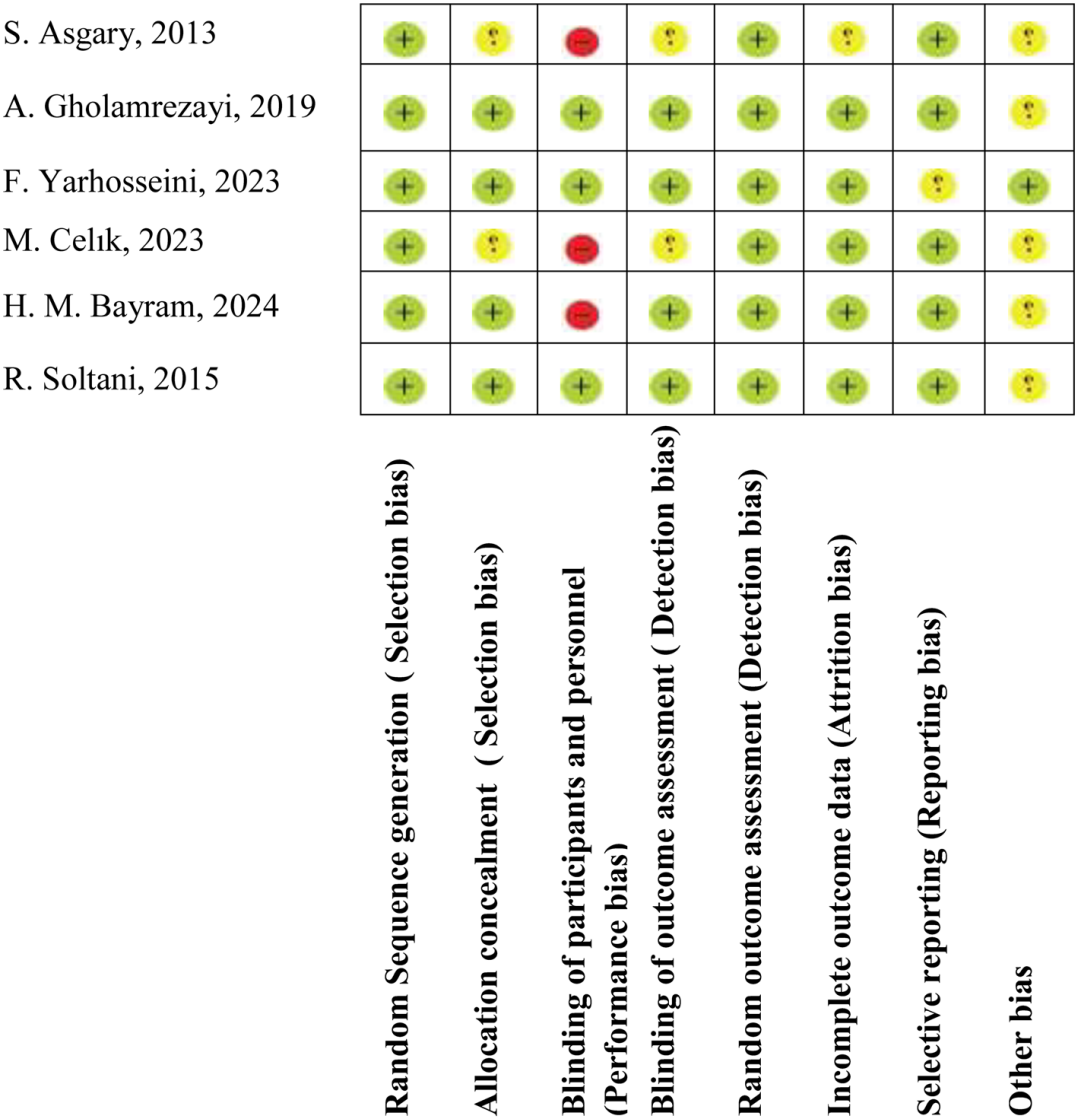
Results of quality assessment of included studies using Cochrane Collaboration's risk of bias tool. (+) Low risk of bias. (−) High risk of bias. (?) Unclear risk of bias.

The main characteristics of studies included can be seen in Table 1. Records were published between 2013 and 2024. Of these 6 studies, 4 were performed in Iran (Asgary, Kelishadi, et al. 2013; Gholamrezayi et al. 2019; Soltani et al. 2015; Yarhosseini, Sangouni, Sangsefidi, et al. 2023) and two in Turkey (Bayram and Ozturkcan 2020; Celik et al. 2023). All of the studies were randomized and 3 of the studies were double‐blind placebo‐controlled RCTs (Gholamrezayi et al. 2019; Soltani et al. 2015; Yarhosseini, Sangouni, Sangsefidi, et al. 2023). Study populations differed from insulin resistance (Celik et al. 2023), NAFLD (Yarhosseini, Sangouni, Sangsefidi, et al. 2023), MAFLD (Bayram et al. 2024), and dyslipidemia (Asgary, Kelishadi, et al. 2013) to menopause (Gholamrezayi et al. 2019). In three studies (Asgary, Kelishadi, et al. 2013; Bayram et al. 2024; Yarhosseini, Sangouni, Sangsefidi, et al. 2023) the population was male and female, but in two studies (Celik et al. 2023; Gholamrezayi et al. 2019), the study population was only female. The sample size ranged from 40 (Asgary, Kelishadi, et al. 2013) to 84 (Celik et al. 2023). In addition, the age range differed from 9 (Asgary, Kelishadi, et al. 2013) to 65 (Bayram et al. 2024; Yarhosseini, Sangouni, Sangsefidi, et al. 2023) years. The intervention dose was 20 g lyophilized dried CM with or without medical nutrition therapy (MNT) (Celik et al. 2023), 20 cc CM fruit extract (Yarhosseini, Sangouni, Sangsefidi, et al. 2023), 30 g lyophilized dried CM with or without medical nutrition therapy (MNT) (Bayram et al. 2024), 100 g (50 g twice a day) CM L. fresh fruits (Asgary, Kelishadi, et al. 2013), and 900 mg (3 capsules of 300 mg) CM extract capsule per day (Gholamrezayi et al. 2019). Intervention durations were 6 (Asgary, Kelishadi, et al. 2013), 8 (Gholamrezayi et al. 2019) and 12 weeks (Bayram et al. 2024; Celik et al. 2023; Yarhosseini, Sangouni, Sangse, et al. 2023). Based on the grade quality assessment, most of the variables gained moderate quality which attenuates the quality of studies (Figure 2).

**TABLE 1:** Study characteristics of included studies.

First author, year of publication	Country	Study type	Randomized	Study population	Gender	Sample size; intervention arms of measured outcomes	Sample size; control arm	Age range (mean age)	Intervention groups dose	Control group	Supplement form	Duration of intervention (week)	Findings
M. Celık, 2023	Turkey	RCT	Randomized	Insulin resistance	F	64 (22, 21, 21)/group	20	18–45	MNT + 20 g, MNT, 20 g lyophilized dried CM	nothing	Lyophilized dried CM	12	MNT + 20 g CM: ↓BW, ↓BMI, ↓WC, ↓HC, ↓%BF 20 g CM: ↓WC, ↓HC, ↔BW, ↔BMI, ↔%BF
F. Yarhosseini, 2023	Iran	DBPC—RCT	Randomized	NAFLD	f/m	25	25	25–65	20 cc/d	20 cc/d placebo	*C. mas* L. fruit extract	12	↔WC, ↔BW, ↔%BF, ↔ WHR, ↔HC
H. M. Bayram, 2024	Turkey	RCT	Randomized	MAFLD	f/m	44 (21, 22, 22)/group	43	18–65	MNT + 30 g, MNT, 30 g lyophilized dried CM	Nothing (NAFLD and non‐NAFLD cases)	lyophilized CM fruit powder	8	MNT + 30 g CM: ↓BW, ↓BMI, ↓WC, ↓HC, ↓BFM 30 g CM: ↓BW, ↓BMI, ↓WC, ↓HC, ↓BFM
S. Asgary, 2013	Iran	RCT	Randomized	Dyslipidemic children and adolescent	f/m	20	20	9–16	100 g/day (50 g/day twice a day)	nothing	*Cornus mas* L. fresh fruits	6	↔ WHR, ↔BMI
A. Gholamrezayi, 2019	Iran	DBPC—RCT	Randomized	Menopause women	f	42	42	45–60	900 mg/day (3 capsules of 300 mg/day)	900 mg/day starch powder capsules	*Cornus mas* extract capsule	8	↓BW, ↓BMI, ↓WC
R. Soltani, 2015	Iran	DBPC—RCT	Randomized	Type 2 Diabetic Adults	f/m	30	30	41–65	1000 mg/day (2 capsules of 500 mg/day)	2 placebo capsules/day	*Cornus mas* extract capsule	6	↓BMI

### 3.3 Meta‐Analysis Results

#### 3.3.1 Effects of CM Supplementation on BW

**FIGURE 3:**
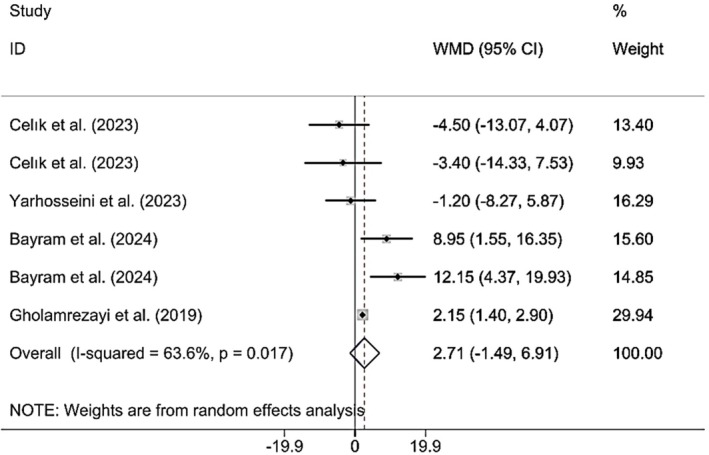
Forest plot presenting mean difference (WMD) and 95% CI for the effect of CM on BW.

Combining the findings of six included RCTs using a random effects model demonstrated that CM administration did not have a significant effect on BW (WMD 2.71 kg; 95% CI (−1.49, 6.91), *p* = 0.206), although a meaningful heterogeneity was observed between the studies included (*I* ^2^ = 63.6%; *p*_heterogeneity_ = 0.017) (Figure 3). However, upon subgroup analysis, it was found that CM supplementation in studies published before the year 2023, on MAFLD and postmenopausal subjects, with a sample size of > 50, where CME capsules were used, the duration was < 12 weeks, and ≥ 30 g of CM was supplemented was significantly increased BW (Table 2). Excluding each individual study using sensitivity analysis did not change the pooled effect size results (Supporting Information S1). Begg's analyses indicated the absence of a small study effect (*p* = 0.573). Additionally, asymmetry was not vivid in the funnel plot (Supporting Information S2).

**TABLE 2:** Subgroup analyses for the effects of CM supplementation on anthropometric indices and body composition.

Group	No. of comparisons	WMD (95% CI)	*p*	*I* ^2^ (%)	*P*‐heterogeneity
BW (Total)	5	2.70 (−1.49, 6.91)	0.206	63.6	0.017
Publication year					
< 2023	1	**2.15 (1.40, 2.89)**	**< 0.001**	0.0	< 0.001
≥ 2023	5	2.73 (−3.96, 9.44)	0.424	70.1	0.009
Population					
Insulin resistance	2	−4.08 (−10.82, 2.66)	0.235	0.0	0.877
NAFLD	1	−1.20 (−8.26, 5.86)	0.739	0.0	< 0.001
MAFLD	2	**10.47 (5.11, 15.83)**	**< 0.001**	0.0	0.559
Postmenopausal	1	**2.15 (1.40, 2.89)**	**< 0.001**	0.0	< 0.001
Gender					
Female	3	0.11 (−4.34, 4.57)	0.959	38.8	0.195
Female and male	3	6.51 (−1.48, 14.51)	0.110	71.4	0.030
Sample size					
≤ 50	3	−2.70 (−7.58, 2.17)	0.277	0.0	0.836
> 50	3	**6.90 (0.04, 13.75)**	**0.049**	78.8	0.009
Supplement form					
Lyophilized dried	4	3.75 (−4.53, 12.04)	0.375	73.5	0.010
CM fruit extract	1	−1.20 (−8.26, 5.86)	0.739	0.0	< 0.001
CME capsule	1	**2.15 (1.40, 2.89)**	**< 0.001**	0.0	< 0.001
Duration (week)					
≥ 12	3	−2.70 (−7.58, 2.17)	0.277	0.0	0.836
< 12	3	**6.90 (0.04, 13.75)**	**0.049**	78.8	0.009
Dose (g)					
< 30	4	0.56 (−2.47, 3.60)	0.716	26.5	0.253
≥ 30	2	**10.47 (5.11, 15.83)**	**< 0.001**	0.0	0.559
BMI (Total)	7	**1.70 (0.16, 3.23)**	**0.030**	88.4	< 0.001
Publication year					
< 2023	3	0.50 (−0.40, 1.40)	0.086	64.1	< 0.001
≥ 2023	4	2.68 (−0.38, 5.76)	0.278	83.6	0.062
Population					
Insulin resistance	2	−0.79 (−3.34, 1.75)	0.542	0.0	0.372
MAFLD	2	**5.15 (3.88, 6.42)**	**< 0.001**	0.0	0.385
Dyslipidemia	1	0.76 (−1.42, 2.94)	0.496	0.0	< 0.001
Postmenopausal	1	**0.94 (0.62, 1.25)**	**< 0.001**	0.0	< 0.001
Type 2 diabetes	1	−0.25 (−1.18, 0.68)	0.601	0.0	< 0.001
Gender					
Female	3	0.59 (−0.63, 1.82)	0.342	21.4	0.280
Female and male	4	2.69 (−0.44, 5.83)	0.093	93.5	< 0.001
Sample size					
≤ 50	3	0.10 (−1.55, 1.76)	0.904	0.0	0.445
> 50	4	**2.57 (0.54, 4.59)**	**0.013**	93.8	3.805
Supplement form					
Lyophilized dried	4	2.68 (−0.38, 5.76)	0.086	83.6	< 0.001
CM fruit extract	1	0.76 (−1.42, 2.94)	0.496	0.0	< 0.001
CME capsule	2	0.43 (−0.72, 1.58)	0.466	82	0.018
Duration (week)					
≥ 12	2	−0.79 (−3.34, 1.75)	0.542	0.0	0.372
< 12	5	**2.24 (0.50, 3.98)**	**0.011**	91.8	< 0.001
Dose (g)					
< 30	4	0.26 (−0.77, 1.30)	0.618	61.9	0.049
≥ 30	3	**3.77 (1.02, 6.53)**	**0.007**	83.8	0.002
BF% (Total)	5	1.45 (−2.49, 5.39)	0.470	81.7	< 0.001
Publication year					
2023	3	−1.59 (−3.54, 0.34)	0.108	0.0	0.650
2024	2	**6.82 (3.79, 9.86)**	**< 0.001**	0.0	0.829
Population					
Insulin resistance	2	−1.15 (−3.33, 1.03)	0.302	0.0	0.763
NAFLD	1	−3.30 (−7.56, 0.96)	0.302	0.0	< 0.001
MAFLD	2	**6.82 (3.79, 9.86)**	**< 0.001**	0.0	0.829
Gender					
Female	2	−1.15 (−3.33, 1.03)	0.302	0.0	0.763
Female and male	3	3.44 (−3.19, 10.08)	0.310	86.1	0.001
Sample size					
≤ 50	3	−1.59 (−3.54, 0.34)	0.108	0.0	0.650
> 50	2	**6.82 (3.79, 9.86)**	**< 0.001**	0.0	0.829
Supplement form					
Lyophilized dried	4	**2.58 (−1.86, 7.03)**	**0.001**	83.0	0.255
CM fruit extract	1	−3.30 (−7.56, 0.96)	0.0	0.0	0.130
Dose					
< 30	3	−1.59 (−3.54, 0.34)	0.108	0.0	0.650
≥ 30	2	**6.82 (3.79, 9.86)**	**< 0.001**	0.0	0.829
Duration					
≥ 12	3	−1.59 (−3.54, 0.34)	0.108	0.0	0.650
< 12	2	**6.82 (3.79, 9.86)**	**< 0.001**	0.0	0.829
BFM (Total)	5	4.01 (−3.27, 11.31)	0.281	88.8	< 0.001
2023	1	−3.40 (−7.6, 0.83)	0.115	0.0	< 0.001
2024	2	**7.51 (4.55, 10.47)**	**< 0.001**	0.0	0.403
Population					
NAFLD	1	−3.40 (−7.63, 0.83)	0.115	0.0	< 0.001
MAFLD	2	**7.51 (4.55, 10.47)**	**< 0.001**	0.0	0.403
Sample size					
≤ 50	1	−3.40 (−7.63, 0.83)	0.115	0.0	< 0.001
> 50	2	**7.51 (4.55, 10.47)**	**< 0.001**	0.0	0.403
Supplement form					
CM fruit extract	1	−3.40 (−7.63, 0.83)	0.115	0.0	< 0.001
Lyophilized CM fruit	2	**7.51 (4.55, 10.47)**	**< 0.001**	0.0	0.403
Dose					
< 30	1	−3.40 (−7.63, 0.83)	0.115	0.0	< 0.001
≥ 30	2	**7.51 (4.55, 10.47)**	**< 0.001**	0.0	0.403
Duration					
≥ 12	1	−3.40 (−7.63, 0.83)	0.115	0.0	< 0.001
< 12	2	**7.51 (4.55, 10.47)**	**< 0.001**	0.0	0.403
WC (Total)	5	0.38 (−5.02, 5.78)	0.890	87	< 0.001
Year of publication					
≥ 2023	5	0.48 (−8.26, 9.22)	0.914	89.1	< 0.001
< 2023	1	−0.52 (−1.20, 0.16)	0.134	0.0	< 0.001
Population					
Insulin resistance	2	−7.94 (−17.74, 1.85)	0.112	72	0.059
NAFLD	1	−3.70 (−9.43, 2.03)	0.206	0.0	< 0.001
MAFLD	2	**10.55 (6.31, 14.79)**	**< 0.001**	0.0	0.738
Postmenopausal	1	−0.52 (−1.20, 0.16)	0.134	0.0	< 0.001
Gender					
Female	3	−4.84 (−12.03, 2.33)	0.186	81.9	0.004
Female and male	3	5.85 (−3.74, 15.45)	0.232	87.0	< 0.001
Sample size					
≤ 50	3	−6.31 (−12.28, −0.35)	0.038	57.1	0.097
> 50	3	6.56 (−2.40, 15.52)	0.151	92.2	< 0.001
Supplement form					
Lyophilized dried	4	1.52 (−9.47, 12.52)	0.786	90.8	< 0.001
CM fruit extract	1	−3.70 (−9.43, 2.03)	0.206	0.0	< 0.001
CME capsule	1	−0.52 (−1.20, 0.16)	0.134	0.0	< 0.001
Dose					
< 30	4	−4.26 (−9.23, 0.70)	0.092	75.3	0.007
≥ 30	2	**10.55 (6.31, 14.79)**	**< 0.001**	0.0	0.738
Duration					
≥ 12	3	**−6.31 (−12.28, −0.35)**	**0.038**	57.1	0.097
< 12	3	6.56 (−2.40, 15.52)	0.151	92.2	< 0.001
HC (Total)	5	1.47 (−4.17, 7.12)	0.608	85.9	< 0.001
Year of publication					
2023	3	**−3.14 (−5.63, −0.65)**	**0.013**	0.0	0.852
2024	2	**7.25 (4.23, 10.26)**	**< 0.001**	0.0	0.326
Population					
Insulin resistance	2	−2.95 (−8.20, 2.30)	0.271	0.0	0.576
NAFLD	1	**−3.20 (−6.02, −0.37)**	**0.027**	0.0	< 0.001
MAFLD	2	**7.25 (4.23, 10.26)**	**< 0.001**	0.0	0.326
Gender					
Female	2	−2.95 (−8.20, −8.20)	0.271	0.0	0.576
Female and male	3	3.85 (−3.93,11.64)	0.332	92.2	< 0.001
Sample size					
≤ 50	3	**−3.14 (−5.63, −0.65)**	**0.013**	0.0	0.852
> 50	2	**7.25 (4.23, 10.26)**	**< 0.001**	0.0	0.326
Supplement form					
Lyophilized dried	4	3.02 (−2.64, 8.70)	0.296	75.3	0.007
CM extract	1	**−3.20 (−6.02, −0.37)**	**0.027**	0.0	< 0.001
Dose					
< 30	3	**−3.14 (−5.63, −0.65)**	**0.013**	0.0	0.852
≥ 30	2	**7.25 (4.23, 10.26)**	**< 0.001**	0.0	0.326
Duration					
≥ 12	3	**−3.14 (−5.63, −0.65)**	**0.013**	0.0	0.852
< 12	2	**7.25 (4.23, 10.26)**	**< 0.001**	0.0	0.326

*Note:* All bold values are significant.

#### 3.3.2 Effects of CM Supplementation on BMI Change

**FIGURE 4:**
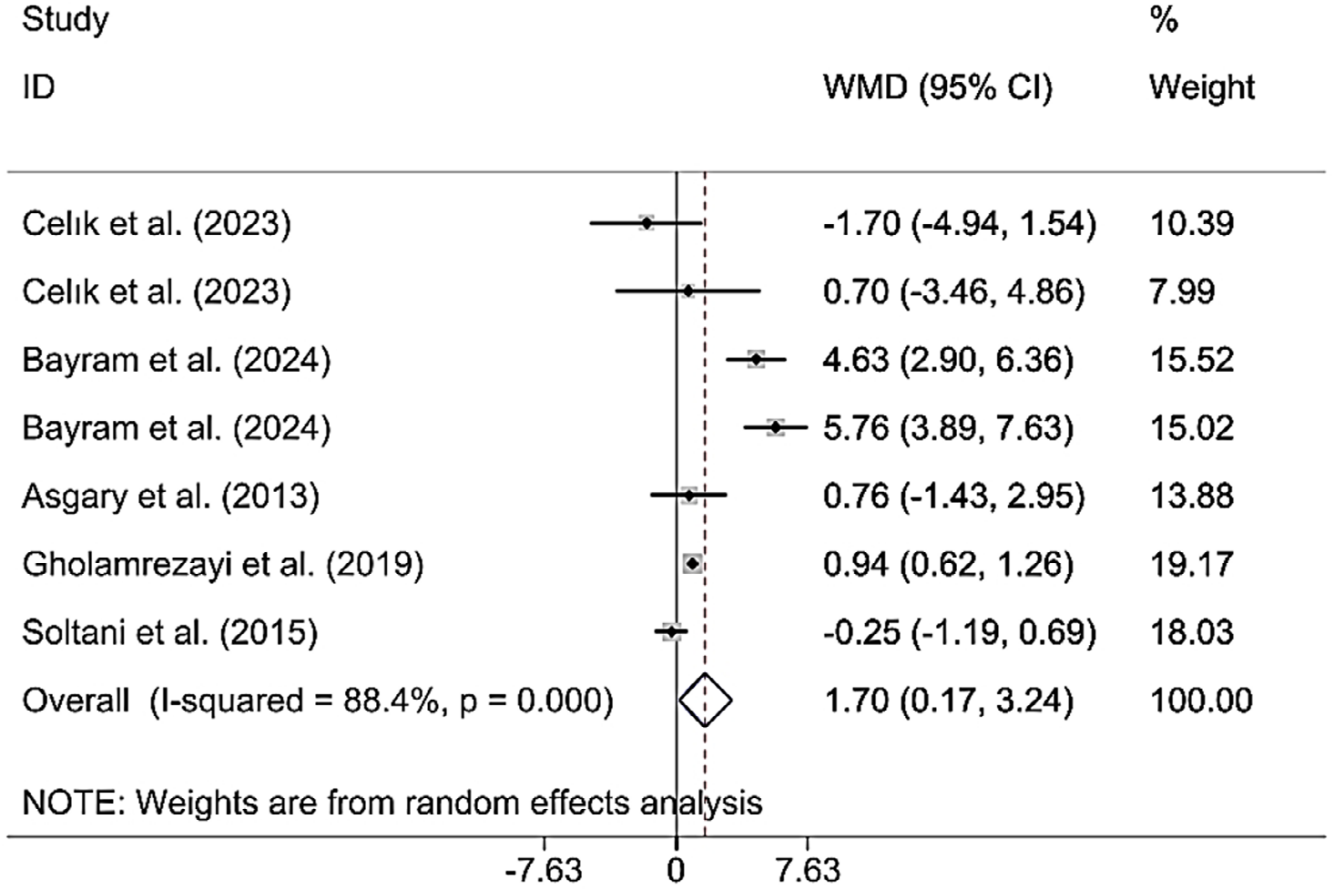
Forest plot presenting mean difference (WMD) and 95% CI for the effect of CM on BMI.

Combining the findings of six included RCTs using a random effects model verified that CM administration had a significant effect on BMI (WMD 1.70 kg/m^2^; 95% CI (0.17, 3.24), *p* = 0.030), despite significant heterogeneity (*I* ^2^ = 88.4%; *p*_heterogeneity_ < 0.001) being observed (Figure 4). However, upon subgroup analysis, it was found that CM supplementation among MAFLD and postmenopausal patients, individuals > 50 years, and the intervention duration was < 12 weeks (Table 2). Excluding each individual study using sensitivity analysis did not change the pooled effect size results (Supporting Information S1). Begg's analyses indicated the absence of a small study effect (*p* = 0.652). Additionally, asymmetry was not vivid in the funnel plot (Supporting Information S2).

#### 3.3.3 Effects of CM Supplementation on WC Change

**FIGURE 5:**
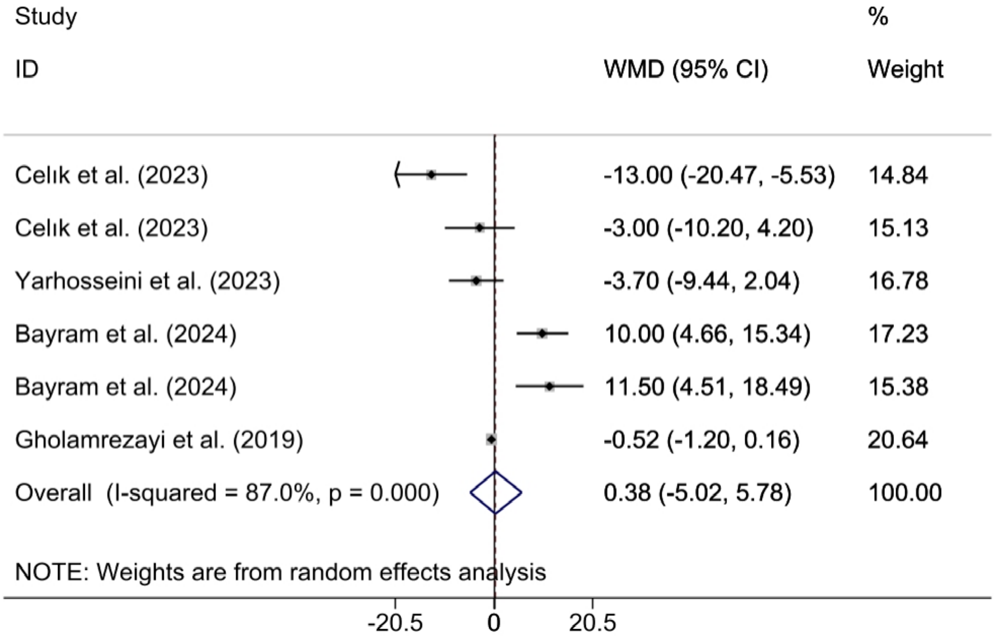
Forest plot presenting mean difference (WMD) and 95% CI for the effect of CM on WC.

Combining the findings of 6 included RCTs using a random effects model demonstrated that CM administration was not significant on WC (WMD 0.38 cm; 95% CI (−5.02, 5.78), *p* = 0.890), despite high heterogeneity (*I* ^2^ = 87%; *p*_heterogeneity_ < 0.001) being observed (Figure 5). However, upon subgroup analysis, it was found that CM supplementation in studies where the study population was MAFLD patients, duration was ≥ 12, and doses were ≥ 30 g (Table 2). Excluding each individual study using sensitivity analysis did not change the pooled effect size results (Supporting Information S1). Begg's analyses indicated the absence of a small study effect (*p* = 0.573). Additionally, asymmetry was not vivid in the funnel plot (Supporting Information S2).

#### 3.3.4 Effects of CM Supplementation on HC Change

**FIGURE 6:**
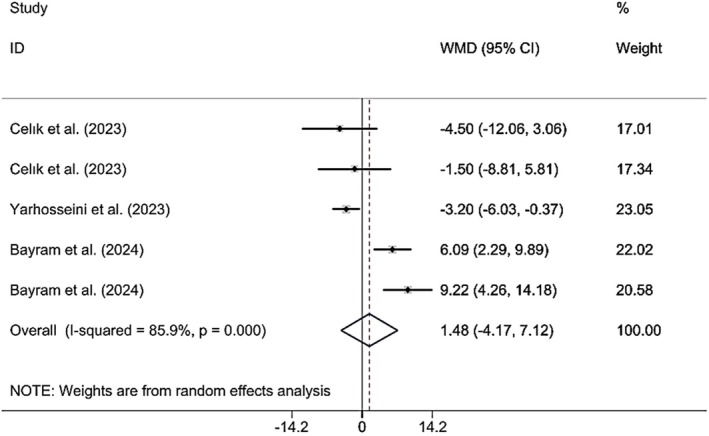
Forest plot presenting mean difference (WMD) and 95% CI for the effect of CM on HC.

Combining the findings of 6 included RCTs using a random effect model demonstrated that CM administration did not have a significant effect on HC (WMD 1.47 cm; 95% CI (−4.17, 7.12), *p* = 0.608), although a considerable heterogeneity among the included studies (*I* ^2^ = 85.9%; *p*_heterogeneity_ < 0.001) was observed (Figure 6). However, upon subgroup analysis, it was found that CM supplementation in studies where the study population was MAFLD and NAFLD patients and CM fruit extract was supplemented yielded significant findings (Table 2). Excluding each individual study using sensitivity analysis did not result in any significant difference in the pooled effect size (Supporting Information S1). Begg's analyses indicated the absence of a small study effect (*p* = 1). Additionally, there was no evidence of asymmetry in the funnel plot (Supporting Information S2).

#### 3.3.5 Effects of CM Supplementation on BF%

**FIGURE 7:**
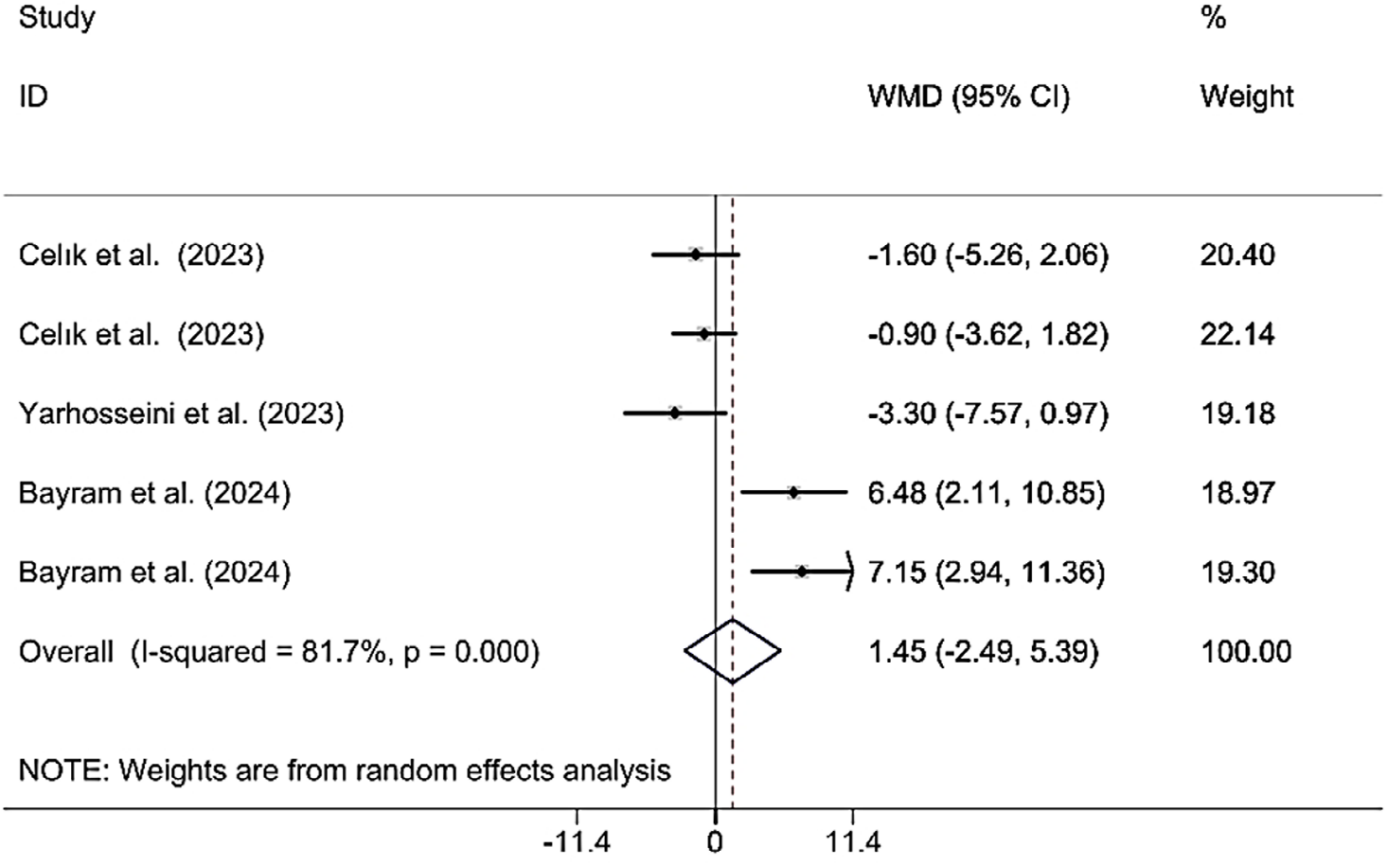
Forest plot presenting mean difference (WMD) and 95% CI for the effect of CM on BF%.

Combining the findings of 6 included RCTs using a random effect model demonstrated that CM administration did not have a significant effect on BF% (WMD 1.45%; 95% CI (−2.49, 5.39), *p* = 0.470), despite significant heterogeneity (*I* ^2^ = 81.7%; *p*_heterogeneity_ < 0.001) being observed (Figure 7). However, upon subgroup analysis, it was found that CM supplementation in studies published in 2024, the population was MAFLD patients, the sample size was > 50, the lyophilized dried form was used, doses were ≥ 30 g, and the duration was < 12 weeks, resulted in significant findings (Table 2). Excluding each individual study using sensitivity analysis did not change the pooled effect size (Supporting Information S1). Begg's analyses indicated the absence of a small study effect (*p* = 0.624). Additionally, no evidence of asymmetry in the funnel plot was detected (Supporting Information S2).

#### 3.3.6 Effects of CM Supplementation on FM

**FIGURE 8:**
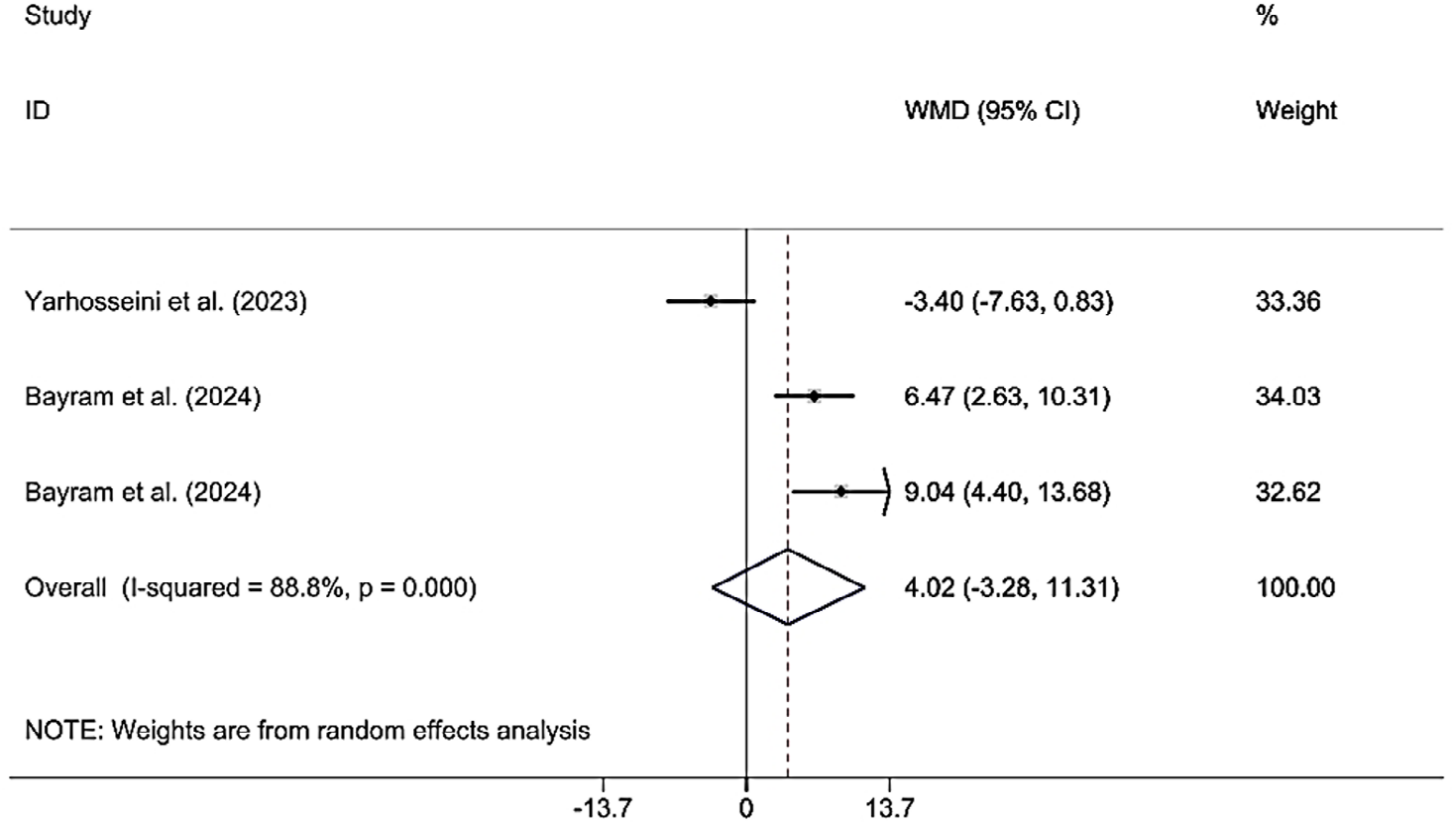
Forest plot presenting mean difference (WMD) and 95% CI for the effect of CM on FM.

Combining the findings of six included RCTs using a random effect model demonstrated that CM administration did not have a significant effect on FM (WMD 4.01 kg; 95% CI (−3.28, 11.31), *p* = 0.281), although high heterogeneity (*I* ^2^ = 88.8%; *p*_heterogeneity_ < 0.001) was observed (Figure 8). However, upon subgroup analysis, it was determined that CM supplementation in studies published in 2024, supplementation was accomplished on MAFLD patients, sample size was > 50, the lyophilized dried form was used, doses were ≥ 30 g, and the duration was < 12 weeks; findings were significant (Table 2). Excluding each individual study using sensitivity analysis did not change the pooled effect size (Supporting Information S1). Begg's analyses indicated the absence of a small study effect (*p* = 0.602). Additionally, asymmetry was not found in the funnel plot (Supporting Information S2).

## 4 Discussion

The global prevalence of obesity has risen dramatically, posing a significant public health challenge due to its association with metabolic disorders such as T2DM, CVD, and NAFLD (Zhang et al. 2024). CM, rich in polyphenolics such as iridoids, anthocyanins, and flavonols, has demonstrated promising anti‐obesity effects in preclinical and clinical studies (Dzydzan et al. 2022). Research suggests that its polyphenolics modulate adipogenesis, improve insulin sensitivity, and reduce oxidative stress and inflammation—key pathways implicated in obesity‐related metabolic dysfunction (Danielewski et al. 2024).

While preclinical studies suggest CM has anti‐obesity potential, the findings of the present systematic review and meta‐analysis exhibited no significant effect for CM administration on BW, BF%, FM, WC, and HC. While a significant increase was observed in the case of BMI. Nevertheless, CM administration was significantly efficient in enhancing BW, BMI, BF%, FM, WC, and HC among MAPLD patients, studies with > 50 sample sizes, the lyophilized dried and CM fruit extract forms were used, intervention duration was < 12 weeks, and doses were ≥ 30 g.

Despite the insignificant results observed for CM administration on anthropometric indices and body composition in the current meta‐analysis, few studies reported significant reductions (Bayram et al. 2024; Celik et al. 2023; Gholamrezayi et al. 2019). The anti‐obesity effects of CM are mediated through the mitogen‐activated protein kinase (MAPK) and Nuclear Factor Kappa B (NF‐κB) signaling pathways, resulting in a decrease in inflammation, promoting cellular protection, and potentially aiding in obesity reduction. Moreover, phenolic compounds such as anthocyanins affect adipose tissue by altering adipocytokine expression and increasing adiponectin levels. Also, by constraining pancreatic lipase activity, anthocyanins decline fat absorption in the gut and prevent visceral fat accumulation (Azzini et al. 2017). Moreover, evidence suggests that CM and its main compounds inhibit hepatic lipogenesis, increase hepatic lipid oxidation and clearance, regulate the expression of peroxisome proliferator‐activated receptors (PPARs), increase the activity of the AMP‐activated protein kinase (AMPK) pathway in the white adipose tissue, and activate pancreatic lipase and lipid absorption (Yarhosseini, Sangouni, Sangse, et al. 2023).

In fact, in the mentioned studies, (Bayram et al. 2024; Celik et al. 2023; Gholamrezayi et al. 2019), CM was administered alongside diet and physical activity, which provide a synergistic effect in weight reduction. Another study (Jayaprakasam et al. 2006) assessed the effect of anthocyanin and peroric acid obtained from CM alongside a high‐fat diet, which resulted in a meaningful reduction in weight gain in the anthocyanin group compared to the control group. Moreover, the lyophilized dried fruit form of CM was used, which is acknowledged as the superior form for preserving antioxidants and was also standardized based on the carbohydrate and total anthocyanin content of the fruit. Also, food consumption records were gathered as participants' self‐reports, thereby increasing the probability of reporting less than actual intake (Bayram et al. 2024; Celik et al. 2023).

The limited number of RCTs assessing the pure effects of anthocyanin obtained from CM may explain the insignificant findings observed. Differences in health status of participants, duration of follow‐up, type of supplement, and dosage of supplement are other considerable factors (Asgary, Kelishadi, et al. 2013; Yarhosseini, Sangouni, Sangse, et al. 2023). Furthermore, studies were mainly accomplished in Iran and Turkey with Asian body composition, high prevalence of obesity, and metabolic disorders. This also may explain the significant augment of BMI, since it is a measure of body fat based on height, weight, race, and potentially effect energy expenditure and overall body composition (Molani‐Gol and Rafraf 2024). Additionally, body composition and anthropometric indices exhibit significant variations between genders due to differences in hormonal profiles, fat distribution, and lean mass accumulation. Men typically have higher lean body mass, and women tend to have higher fat mass due to variances in testosterone and estrogen levels. Men generally show android obesity, linked to visceral fat and metabolic risks, whereas women exhibit gynoid fat distribution (Karastergiou et al. 2012). Also, while BMI thresholds are gender‐neutral, women often have higher fat mass at the same BMI compared to men (Rothman 2008). Accordingly, the study population of studies included in the current meta‐analysis was complex, consisting of both genders (Asgary, Kelishadi, et al. 2013; Bayram et al. 2024; Soltani et al. 2015; Yarhosseini, Sangouni, Sangsefidi, et al. 2023) or female only (Celik et al. 2023; Gholamrezayi et al. 2019). In fact, due to the broad age range, a considerable number of women were menopausal, such as Gholamrezayi et al.'s study, which were only menopausal women (Gholamrezayi et al. 2019). Another important matter that must be considered is that body composition and anthropometric indices must be assessed simultaneously in order to fully assess the effect of CM administration. In few studies (Asgary, Kelishadi, et al. 2013; Gholamrezayi et al. 2019; Soltani et al. 2015), not all of the indices were measured, leaving doubt regarding the results of other measures.

Subgroup analyses revealed interesting results. Based on subgroup analyses, CM fruit administration significantly enhanced BW, BMI, BF%, FM, WC, and HC when supplementation was accomplished on MAPLD patients, the sample size was > 50, the lyophilized dried and CM fruit extract forms were used, the intervention duration was < 12 weeks, and doses were ≥ 30 g, suggesting significant reductions, possibly in longer durations and higher doses.

The current systematic review and meta‐analysis had multiple limitations and advantages. The first limitation was the small number of studies included. This is because we narrowed it to CM fruit administration, while other studies generally consider various anthocyanin‐rich sources such as blueberries, raspberries, and cranberry as CM. Another limitation was geographical and population heterogeneity (Iran and Turkey), which limited the extrapolation of findings to other populations with varying genetic, dietary, and lifestyle backgrounds. Variability in reporting body composition metrics may have influenced pooled estimates. Furthermore, assessing anthropometric indices without metabolic factors may not provide a thorough feature of the effectiveness of CM on body composition. For future research, it is advised to thoroughly assess the effectiveness of CM on health. Excluding gray or non‐English literature was another limitation of the study. Moreover, the study protocol was not registered in PROSPERO, which may potentiate bias. Hence, for further research, it is suggested to establish well‐designed high‐quality randomized controlled trials in different regions and various health conditions in order to fully assess its mechanism of action. The advantages of the study were including well‐designed RCTs, assessing anthropometric indices and body composition together, and evaluating the quality of the studies. Based on the grade quality assessment, most of the variables gained moderate quality, which attenuates the quality of studies.

## 5 Conclusion

The findings of the present systematic review and meta‐analysis indicated a significant enhancement in BMI, and no significant changes in BW, BF%, FM, WC, and HC after administering CM. Although CM exhibits favorable metabolic properties in preclinical and clinical studies, current evidence does not support its efficacy as a standalone intervention for anthropometric improvement. Consequently, a diversified dietary approach incorporating multiple anthocyanin‐rich food sources—combined with evidence‐based nutritional strategies and regular physical activity—is recommended to optimize metabolic benefits and body composition outcomes. Hence, future research should prioritize well‐designed, long‐term follow‐up RCTs investigating CM as an adjunct therapy to multimodal lifestyle interventions, focusing on both anthropometric indices and biochemical markers.

## Author Contributions

**Faezeh Ghalichi:** conceptualization (equal), data curation (equal), formal analysis (equal), investigation (equal), methodology (supporting), project administration (equal), supervision (equal), validation (equal), visualization (equal), writing – original draft (supporting), writing – review and editing (equal). **Roghayeh Molani‐Gol:** formal analysis (equal), methodology (equal), software (equal), writing – original draft (equal), writing – review and editing (equal). **Neda Ehsani:** conceptualization (equal), data curation (equal), writing – original draft (equal). **Yaser Khajebishak:** investigation (equal), project administration (equal), supervision (equal), writing – original draft (equal).

## Conflicts of Interest

The authors declare no conflicts of interest.

## Supporting information


Data S1.

Supplementary S1.

Supplementary S2.

Table S1.

Table S2.

## Data Availability

The data that support the findings of this study are available on request from the corresponding author. The data are not publicly available due to privacy or ethical restrictions.
