# Supplementary material for: A Systematic Review and Meta‐Analysis of the Impact of Cornus mas L. on Anthropometric Indices and Body Composition
Source: Food Sci Nutr. 2025 Jul 15;13(7):e70404. doi: 10.1002/fsn3.70404 (PMC12261034; doi:10.1002/fsn3.70404)
Supplement: Supplementary file 2 — Supplementary S1. [file FSN3-13-e70404-s005.docx]

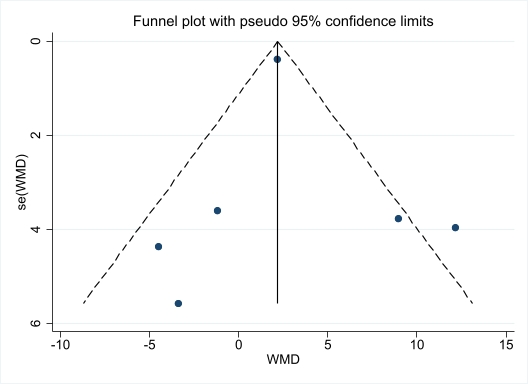


**Figure 1. Funnel plot presenting mean difference (WMD) and 95% CI for the effect of CM on BW**


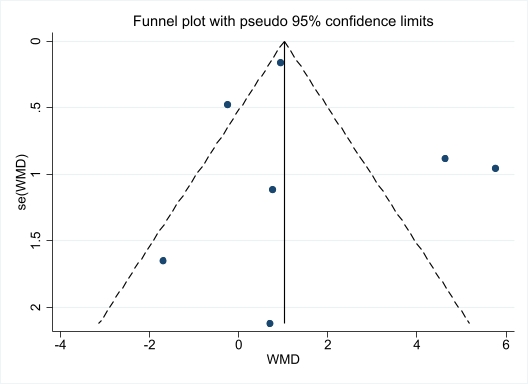


**Figure 2. Funnel plot presenting mean difference (WMD) and 95% CI for the effect of CM on BMI**


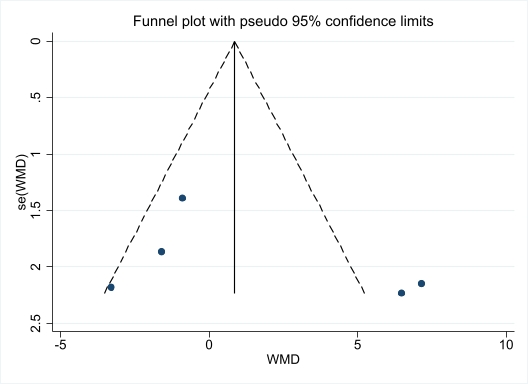


**Figure 3. Funnel plot presenting mean difference (WMD) and 95% CI for the effect of CM on BF%**


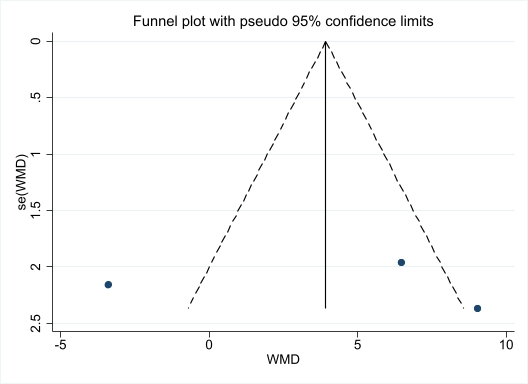


**Figure 4. Funnel plot presenting mean difference (WMD) and 95% CI for the effect of CM on FM**


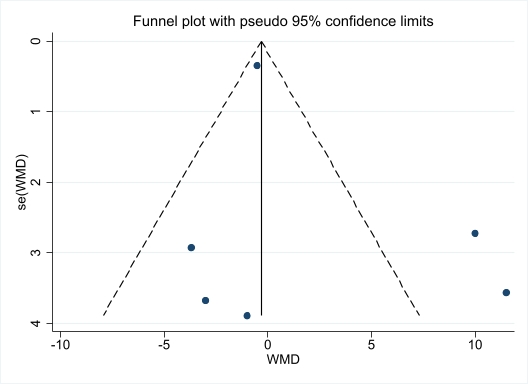


**Figure 5. Funnel plot presenting mean difference (WMD) and 95% CI for the effect of CM on WC**


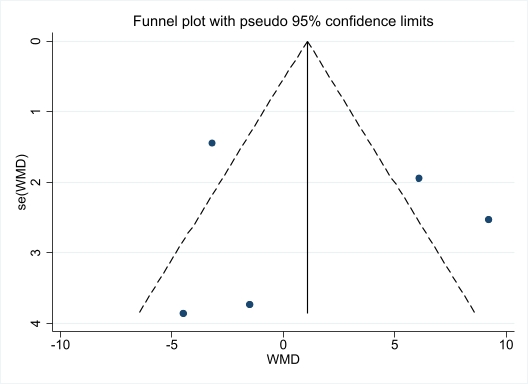


**Figure 6. Funnel plot presenting mean difference (WMD) and 95% CI for the effect of CM on HC**
