# Supplementary material for: A Systematic Review and Meta‐Analysis of the Impact of Cornus mas L. on Anthropometric Indices and Body Composition
Source: Food Sci Nutr. 2025 Jul 15;13(7):e70404. doi: 10.1002/fsn3.70404 (PMC12261034; doi:10.1002/fsn3.70404)
Supplement: Supplementary file 4 — Table S1. [file FSN3-13-e70404-s003.docx]

Table 1. Sensitive literature search strategy based on database filters

| **Database** | **Search items** |
| --- | --- |
| **PubMed** | **(((((cornelian cherry[Title/Abstract]) OR (cornelian mass[Title/Abstract])) OR (Cornus mas[Title/Abstract])) OR ("Cornus"[Mesh])) AND ((((((((((((((((((weight[Title/Abstract]) OR (Body mass index[Title/Abstract])) OR (BMI[Title/Abstract])) OR (waist circumference[Title/Abstract])) OR (WC[Title/Abstract])) OR (Hip circumference[Title/Abstract])) OR (HC[Title/Abstract])) OR (waist-to-hip ratio[Title/Abstract])) OR (WHR[Title/Abstract])) OR (body fat mass[Title/Abstract])) OR (BFM[Title/Abstract])) OR (body fat percent[Title/Abstract])) OR (BFP[Title/Abstract])) OR (fat free mass[Title/Abstract])) OR (FFM[Title/Abstract])) OR (muscle mass[Title/Abstract])) OR (MM[Title/Abstract])) OR (((("Body Weight"[Mesh]) OR "Body Mass Index"[Mesh]) OR "Waist Circumference"[Mesh]) OR "Waist-Hip Ratio"[Mesh]))) AND (((((((randomized controlled trial[Publication Type]) OR (controlled clinical trial[Publication Type])) OR (trial[Title/Abstract])) OR (clinical trial[Title/Abstract])) OR (supplementation[Title/Abstract])) OR (random*[Title/Abstract])) OR ("Randomized Controlled Trial" [Publication Type] OR "Controlled Clinical Trial" [Publication Type]))** |
| **Scopus** | **( ( ALL ( cornelian AND cherry ) OR TITLE-ABS-KEY ( cornelian AND mass ) OR TITLE-ABS-KEY ( cornus AND mas ) ) ) AND ( ( ALL ( weight ) OR TITLE-ABS-KEY ( body AND mass AND index ) OR TITLE-ABS-KEY ( BMI ) OR TITLE-ABS-KEY ( waist AND circumference ) OR TITLE-ABS-KEY ( WC ) OR TITLE-ABS-KEY ( hip AND circumference ) OR TITLE-ABS-KEY ( HP ) OR TITLE-ABS-KEY ( waist-to-hip AND ratio ) OR TITLE-ABS-KEY ( WHR ) AND TITLE-ABS-KEY ( body AND fat AND mass ) OR TITLE-ABS-KEY ( body AND fat AND percent ) OR TITLE-ABS-KEY ( BFP ) OR TITLE-ABS-KEY ( fat AND free AND mass ) OR TITLE-ABS-KEY ( FFM ) OR TITLE-ABS-KEY ( muscle AND mass ) OR TITLE-ABS-KEY ( MM ) ) ) AND ( ( ALL ( randomized AND controlled AND trial ) OR TITLE-ABS-KEY ( body AND mass AND index ) OR TITLE-ABS-KEY ( controlled AND clinical AND trial ) OR TITLE-ABS-KEY ( supplementation ) OR TITLE-ABS-KEY ( clinical AND trial ) OR TITLE-ABS-KEY ( random* ) ) )** |
| **Web of Science** | **((cornelian cherry OR cornelian mass (topic) OR cornus mas (topic) AND weight (topic) OR body mass index (topic) OR BMI (topic) OR waist circumference (topic) OR WC (topic) OR hip circumference (topic) OR HP (topic) OR waist-to-hip ratio (topic) OR WHR (topic) AND body fat mass (topic) OR body fat percent (topic) OR BFP (topic) OR fat free mass (topic) OR FFM (topic) OR muscle mass (topic) OR mm (topic))) AND ((randomized controlled trial (topic) OR controlled clinical trial (topic) OR supplementation (topic) OR clinical trial (topic) OR random* (topic)))** |
