# Supplementary material for: A Systematic Review and Meta‐Analysis of the Impact of Cornus mas L. on Anthropometric Indices and Body Composition
Source: Food Sci Nutr. 2025 Jul 15;13(7):e70404. doi: 10.1002/fsn3.70404 (PMC12261034; doi:10.1002/fsn3.70404)
Supplement: Supplementary file 5 — Table S2. [file FSN3-13-e70404-s004.docx]

**Table 2.** Summary of results and quality of evidence assessment using the GRADE approach

| **Outcome measures** | **Summary of findings** | | High | | | | | |
| --- | --- | --- | --- | --- | --- | --- | --- | --- |
|  | **No. of patients/meta-analyses** | **Effect size (95% CI)** | **Risk of bias ^a^** | **Inconsistency ^b^** | **Indirectness ^c^** | **Indirectness ^d^** | **Publication bias ^e^** | **Quality of evidence ^f^** |
| BW | 305/6 | 2.70 (-1.49, 6.91) | Not serious | Serious | Not serious | Serious | Not serious | Moderate |
| BMI | 355/6 | 1.70 (0.16, 3.23) | Not serious | Serious | Not serious | Serious | Not serious | Moderate |
| BF% | 134/6 | 1.45 (-2.49, 5.39) | Not serious | Serious | Not serious | Serious | Not serious | Moderate |
| FM | 87/6 | 4.01(-3.27, 11.31) | Not serious | Serious | Not serious | Serious | Not serious | Moderate |
| WC | 305/6 | 0.38 (-5.02, 5.78) | Not serious | Serious | Not serious | Serious | Not serious | Moderate |
| HC | 171/6 | 1.47 (-4.17, 7.12) | Not serious | Serious | Not serious | Serious | Not serious | Moderate |

BW, body weight; BMI, body mass index; BF%, body fat percent; FM, fat mass; WC, waist circumference; HC, hip. ^a^ Risk of bias based on the Cochrane Collaboration's risk of bias results. ^b^ Downgraded if there was a substantial unexplained heterogeneity (I^2^ > 50%, P < 0.10) that was unexplained by meta-regression or subgroup analyses. ^c^ Downgraded if there were factors present relating to the participants, interventions, or outcomes that limited the generalizability of the results. ^d^ Downgraded if optimal information size was not met, or the 95% CI include the null value lower and upper bounds of the 95%CI were <0.95 and >1.05, respectively. ^e^ Downgraded if there was an evidence of publication bias using funnel plot. ^f^ Since all included studies were meta-analyses of randomized clinical trials, the certainty of the evidence was graded as high for all outcomes by default and then downgraded based on prespecified criteria. Quality was graded as high, moderate, low, very low
